# Supplementary material for: Short-term interval aerobic exercise training does not improve memory functioning in relapsing-remitting multiple sclerosis—a randomized controlled trial
Source: PeerJ. 2018 Dec 12;6:e6037. doi: 10.7717/peerj.6037 (PMC6295157; doi:10.7717/peerj.6037)
Supplement: Supplemental Information 3 [file peerj-06-6037-s003.pdf]

# Protocol AERCONN

## I) Administrative information

**1 Title:** *Descriptive title identifying the study design, population, interventions, and, if applicable, trial acronym*

**Study protocol for a randomized controlled phase IIa trial investigating the effects of a standardized aerobic exercise intervention on cognitive function and brain connectivity in relapsing-remitting multiple sclerosis**

**Trial Acronym:** AERCONN

**German study title:** Einfluss von aerobem Sporttraining auf kognitive Funktionen und Konnektivität bei multipler Sklerose.

**2 Trial Registration**
*2 a: Trial identifier and registry name. If not yet registered, name of intended registry*
Registered at clinicaltrials.gov NCT02005237 (December 3, 2013).

**3 Protocol Version**: *Date and version identifier*
*Version 1 29-08-2013 (CH)*
*Version 2 08-11-2013 (SMG)*
*Version 3 23-11-2013 (SMG)*

**4 Funding:** *Sources and types of financial, material, and other support*
German Ministry of Education and Research through the Biopharma Call “Neu-Quadrat”

Universitätsklinikum Hamburg-Eppendorf

5 Roles and responsibilities

*5a: Names, affiliations, and roles of protocol contributors*

Prof. Dr. med. Christoph Heesen (CH)
MS Day Hospital and Outpatient Unit
Institute for Neuroimmunology and Clinical MS Research and Department of Neurology, University Medical Center Hamburg-Eppendorf
Martinistrasse 52
20246 Hamburg
Tel.: 0049-40 7410-54076
Fax: 0049-40 7410-56973
Email: heesen@uke.de

Dr. phil. Dipl.-Psych. Stefan M. Gold (SMG)
Institute for Neuroimmunology and Clinical MS Research
University Medical Center Hamburg-Eppendorf

Prof. Dr. med. Dr. phil. Karl-Heinz Schulz (KHS)
Institute for Medical Psychology and Excellence Center for Sports Medicine (Athleticum)
University Medical Center Hamburg-Eppendorf

Prof. Dr. med. Andreas K. Engel (AE)
Department of Neurophysiology and Pathophysiology
University Medical Center Hamburg-Eppendorf

Dipl.-Sportwiss. Stefan Patra (SP)
Excellence Center for Sports Medicine (Athleticum)
University Medical Center Hamburg-Eppendorf

Dr. med. Susanne Siemonsen (SS)
Section for Multiple Sclerosis Imaging (SeMSI)
Department of Neuroradiology
University Medical Center Hamburg-Eppendorf

Dr. rer. nat. Guido Nolte (GN)
Department of Neurophysiology and Pathophysiology
University Medical Center Hamburg-Eppendorf

PD Dr. phil. Dipl.-Psych. Iris K. Penner (IKP)
Department of Psychology
University of Basel, Switzerland

Gesche Ketels (GK)
Department of Physiotherapy
University Medical Center Hamburg-Eppendorf

Eik Vetorazzi (EV)
Department of Medical Biometry and Epidemiology
University Medical Center Hamburg-Eppendorf

Dr. med. Sabine Fleischer (SF)
Institute for Neuroimmunology and Clinical MS Research
University Medical Center Hamburg-Eppendorf

Dipl.-Psych. Anja Fischer (AF)
Institute for Neuroimmunology and Clinical MS Research
University Medical Center Hamburg-Eppendorf

Dr. med. Sina Rosenkranz (SR)
MS Day Hospital and Outpatient Unit
Institute for Neuroimmunology and Clinical MS Research and Department of Neurology,
University Medical Center Hamburg-Eppendorf

M.Sc. Arne Ewald (AE)
Department of Neurophysiology and Pathophysiology
University Medical Center Hamburg-Eppendorf

M.Sc. Tom Pfeffer (TP)
Department of Neurophysiology and Pathophysiology
University Medical Center Hamburg-Eppendorf

M.Sc. Sarah Bütof (SB)
Department of Neurophysiology and Pathophysiology
University Medical Center Hamburg-Eppendorf

Dipl. Psych. Marion Höfle (MH)
Department of Neurophysiology and Pathophysiology
University Medical Center Hamburg-Eppendorf

CH, SMG, and KHS developed the overall study protocol with contributions from AKE and GN (functional and structural connectivity), IKP (cognitive measures and task-fMRI), SS (structural MRI), and SP (exercise physiology and training concepts).

CH supervises patient recruitment and clinical assessments. AKE and GN are responsible for MEG outcomes. SF acts as the overall financial project manager and scientific coordinator.

*5b: Name and contact information for the trial sponsor*

**Bundesministerium für Bildung und Forschung**
**VDI**

*5c: Role of study sponsor and funders, if any, in study design; collection, management, analysis, and interpretation of data; writing of the report; and the decision to submit the report for publication, including whether they will have ultimate authority over any of these activities*

The funding body has no influence on the design, administration, analysis and interpretation as well as the dissemination of results of this study.

*5d: Composition, roles, and responsibilities of the coordinating centre, steering committee, endpoint adjudication committee, data management team, and other individuals or groups overseeing the trial, if applicable (see Item 21a for data monitoring committee)*

**Principal Investigators (CH, SMG, AKE, GN, IKP, KHS) and Research Team (AF, SP, GK, SS, SF, EV, AE, TP, SB, MH)**
Design and conduct of AERCONN
Preparation of protocol and revisions
Preparation of CRFs
Publication of study reports

**Steering committee (SC)** (PIs)
Responsibilities: Agreement of final protocol (approved 21-11-2013).
Reviewing progress of study and if necessary approving changes to the protocol and/or investigators brochure to facilitate the conduction of the study.

**Data Manager**
Arne Ewald
Maintenance of trial IT system and data entry
Data verification

**Data Monitoring and Safety Board (DMSB)**
Christoph Heesen, Manuel Friese

# II) Introduction

***6 Background and rationale***

*6a: Description of research question and justification for undertaking the trial, including summary of relevant studies (published and unpublished) examining benefits and harms for each intervention*

Historically, MS patients have been advised to refrain from physical exercise due to heat sensitivity of some symptoms. However, a first randomized controlled trial in 1996 demonstrated that exercise may have beneficial effects on quality of life. Since then, improved quality of life and walking ability in MS after exercise training has been confirmed in several RCTs. Based on preclinical evidence and first pilot studies in humans, this phase IIa will investigate whether exercise has beneficial effects beyond psychological well-being, namely improving cognitive function and related measures of brain connectivity.

Exercise may be "neuroprotective" in MS

Voluntary wheel running enhances hippocampal neurogenesis in animal models (Cotman et al 2007). Importantly, hippocampal damage has been linked to neuropsychiatric symptoms of MS such as depression and memory impairment (Gold et al in press; Gold et al 2010, Sicotte et al 2008; Kiy et al 2011), thereby presenting an important target brain region for neuroprotective therapies in MS. A mechanistic link between exercise and cognitive function has been provided by numerous animal studies indicating increased neuroplasticity and improved cognitive function (Cotman and Berchtold 2002). The underlying neurobiological mechanisms of this protective effect are poorly understood but may include several pathways such as neurotrophic factors (Cotman et al 2007), anti-inflammatory processes (Gleeson et al 2011) and myokines (Pedersen 2011; Pedersen and Febbraio 2012).

Several studies have reported a positive association between aerobic fitness and cognitive parameters in healthy adults (Smith et al 2010; Etnier et al 2006). In addition, exercise interventions appear to enhance cognitive function in both healthy individuals as well as subjects at risk of dementia (Hillman et al 2006; Schulz et al 2012).

Work by Prakash et al (2010, 2011) provided first cross-sectional evidence for differences in gray matter density and resting state networks in trained versus untrained MS patients. Further supporting the biological rationale for CNS effects of exercise in MS,

evidence from the animal model of MS, experimental autoimmune encephalomyelitis (EAE), suggests that exercise might directly affect pathology as some studies indicate disease attenuation through exercise (LePage et al 1996) as well as axonal protection and enhancement of neuronal plasticity (Rossi et al 2009). However, to date for the effectiveness of exercise interventions on neuroprotection merely does not exist. To date, only one study has explored effects of low intensity level exercise on cognitive function in MS patients with minor disability (Oken et al 2006) showing no clear-cut effects. We have recently conducted a pilot trial with n=40 MS patients in the progressive phase (EDSS 4-6) comparing an 8 week (20 session) training period with hand- and bicycle ergometry as well as rowing to a non-exercising waitlist control group. We could demonstrate not only a significant beneficial effect on walking ability but also on neuropsychological tests of attention and verbal learning (Briken et al 2013). In this small trial now provides the basis for studying the effects of exercise on brain structure and function using neuropsychological tests as well as CNS connectivity in the current study.

Novel imaging markers of CNS connectivity

MRI is regarded the most promising tool to assess tissue integrity in the brain in MS. However, due to the slow nature of neurodegenerative processes and the limited sensitivity of conventional MRI surrogate markers, predictive short-term changes have not been established (Barkhof et al 2009). Although non-conventional MRI markers such as T2 subtraction, brain atrophy, DTI or MTR as well as lesion evolution (Barkhof et al 2009; van den Elskamp et al 2010) might detect some aspects of neuroregeneration, until now they seem not sensitive enough for short-term changes.

Measures of functional connectivity such as correlations in spontaneous brain activity provide powerful access to large-scale organizational principles of the central nervous system (Raichle 2010). These analyses may therefore provide a more sensitive tool to detect regeneration and repair. Numerous recent studies have applied fMRI to uncover highly stable resting state and task-related networks in healthy subjects by demonstrating correlated slow fluctuations of the BOLD signal across different brain regions (Raichle 2010; Engel et al. Neuron 2013). Applying this approach to MS patients, a couple of studies have demonstrated changes of coupling in networks relating to cognitive and sensorimotor functions (Roosendaal et al 2010; Faivre et al 2012). And we have recently shown that, in early-stage MS, cognitive decline is associated with increased

resting state coupling despite beginning structural desintegration of cortico-cortical networks (Hawellek et al 2011).

Functional reorganization has been extensively studied in MS by task-specific fMRI with remaining challenges regarding the interpretation of coactivation as compensatory or decompensatory (Filippi and Rocca 2011; Penner et al 2006). Therefore task-fMRI might be a sensitive outcome measure for short-term interventions.

Magnetoencephalography (MEG) provides the best currently available non-invasive method able to capture the spatiotemporal dynamics of brain networks. Using MEG it becomes possible to study local activity and large-scale interactions across a broad range of time scales and in a spectrally resolved manner (Engel et al. Neuron 2013). In particular, changes in power and coherence of fast oscillations at beta-band and gamma-band frequencies can be revealed (Hipp et al 2011) which are known to be closely related to cognitive processing (Engel et al 2001; Womelsdorf et al 2007; Senkowski et al 2008; Siegel et al 2012).

At present, almost nothing is known about the effects of MS pathology on the spatiotemporal CNS network dynamics and no information is available regarding changes over time or in response to an intervention such as exercise.

**7 Objectives:** Specific objectives or hypotheses

This study aims to explore the potential of an aerobic exercise program on brain structure and function in MS in a single-blind, randomized controlled phase IIa trial. We hypothesize that exercise will improve verbal learning and memory (primary endpoint) as well as induce changes in neuroimaging markers of structural and functional CNS connectivity (secondary endpoints). Tertiary outcomes will include walking ability, motor function and coordination, as well as patient-based outcomes (depression, fatigue, and health-related quality of life).

**8 Trial design:** Description of trial design including type of trial (eg, parallel group, crossover, factorial, single group), allocation ratio, and framework (eg, superiority, equivalence, noninferiority, exploratory)

This is a single-blind, randomized, controlled phase IIa trial with a parallel group design comparing 3 months of standardized aerobic exercise training (bicycle ergometry) to a waitlist control group (superiority framework). The allocation ratio of exercise to waitlist control is 1:1. The trial consists of a **primary trial phase** and an **extension phase** (see Figure 1). All primary analyses of endpoints will be restricted to the primary trial phase.

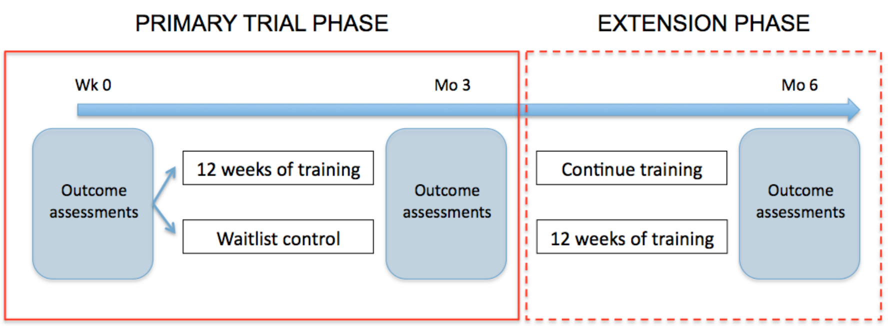


**Figure 1:** Trial Design.

All outcome parameters will be assessed at baseline (week 0), after the intervention (month 3), and at follow-up (month 6), see Figure 1. Each outcome assessment block (blue rectangles) includes: Clinical assessment (neurological scoring and neuropsychological testing), magnetic resonance imaging (structural and functional MRI), magnetencephalography (MEG), and assessments of physical fitness at the Department of Sports Medicine. After completing Month 3 assessments, patients randomized to the WLC will be offered the aerobic exercise training program and patients in the training group will be offered to continue the training free of charge (extension phase).

# III) Methods: Participants, interventions, and outcomes

**9 Study setting:** Description of study settings (e.g., community clinic, academic hospital) and list of countries where data will be collected. Reference to where list of study sites can be obtained

This single-center study will be conducted at the University-Medical Center Hamburg Eppendorf (UKE).

**10 Eligibility criteria:** Inclusion and exclusion criteria for participants. If applicable, eligibility criteria for study centers and individuals who will perform the interventions (e.g., surgeons, psychotherapists)

### 10.1. Patient inclusion criteria

- Relapsing-remitting patients according to McDonald (2010) criteria.
- Currently in remission
- Disease duration < 10 years
- Low to moderate physical disability (EDSS ≤ 3.5)
- On stable immunotherapy > 3 months or without any planned treatment for the next year.

### 10.2. Patient exclusion criteria

- Patients who are not able to understand the study concept due to severe cognitive deficits or psychiatric comorbidity.
- Patients who are currently taking psychotropic drugs
- Patients who are unable to undergo aerobic exercise for medical reasons (e.g. putative or possible heart disease based on screening questionnaire and ECG examination).
- Patients with very active disease, or uncertain stability under current immunotherapy as judged by the treating neurologist (C.H.).
- Patients with implants or body modification (e.g. dental implants, piercings, tattoos, pacemakers etc.) that might interfere with MEG and MRI assessments.
- Patients unable to travel to the study center 2-3 x / week.

### 11 Interventions

*11a: Interventions for each group with sufficient detail to allow replication, including how and when they will be administered*

The intervention implemented is a 3-month aerobic exercise training (bicycle ergometry) tailored to the individual patient's level of aerobic fitness at baseline (spiroergometry). The training program consists of 2-3 sessions per week. The starting point (anchor) of the training program will be determined by the baseline spiroergometry test (see section 11a.1.). The training will follow a pre-specified plan where duration as

well as power (in Watts) will be gradually increased from session to session (see section 11a.2.).

11a.1. Spiroergometry

To determine the individual level of aerobic fitness, all patients will undergo a bicycle spiroergometry using the Metalyser 3b (CORTEX Biophysik GmbH, Leipzig, Germany). This test will be used as the anchor for the individualized training program (see below) as well as to monitor changes in aerobic fitness after the training intervention. The spiroergometry test starts with a resting-phase of 2 minutes. Ergometry will start at 25 Watts and performance will be continuously increased by 1 Watt every 4.8 seconds according to the WHO protocol (resulting in a ramp of 25 Watts/2min). We will continuously obtain stress ECG to monitor cardiovascular function (blood pressure (BP) and heart rate). Oxygen consumption will be recorded using the Metalyser 3b. In addition, blood draws for lactate assessment will be obtained with capillary pipettes (10μl) from one ear every 2 minutes and lactate values quantified by Biosen C-Line (EKF-diagnostic GmbH, Magdeburg, Germany). In addition, subjective exhaustion will be monitored by the Extended Borg scale (6-20) every 2 minutes. Patients will exercise under supervision until exhausted (Borg score of 18-20). Reasons for stopping (f.e. muscular fatigue, dyspnea) will be documented. For safety reasons, ergometry will also be stopped if a patient reaches systolic BP ≥ 200 and/or diastolic BP ≥ 110. If substantial ectopic electric heart activity or other complications occur, the spiroergometry will be stopped and the patient will be excluded from the study. After termination of the exhaustion test, spirometric data will be recorded for another 5 minutes. Lactate will be assessed after 1, 3, and 5 minutes during the cool-down period to detect the maximal lactate value. Two experienced exercise physiologists will determine the thresholds on the plots using semi-automated algorithms and blinded to patient identity. The Lactate thresholds AT and MaxLaSS will be determined using the first uptick in the lactate curve as well as the maximum change in lactate incline. Ventilatory thresholds 1 and 2 will be determined on the ventilation curve and confirmed by curves of ventilatory equivalents for $O_2$ ($VE/VO_2$), $CO_2$ ($VE/VCO_2$), and the slopes of VE and $VCO_2$, respectively (Wasserman et al., 1973). The maximum $VO_2$ value ($ml \cdot kg^{-1} \cdot min^{-1}$) will be obtained close to exhaustion and will be considered as $VO_{2peak}$. The **anchor value** for the individually tailored exercise training will be the power (in Watts) recorded at Extended Borg scale score of 11.

## 11a.2. Individualized training plan

*Training plan overview:* The predefined training structure was designed to achieve a continuous increase of performance from training session (TS) to training session. Each training session consists of 3-5 intervals of training with interspersed breaks. The goal for every TS is to increase performance while keeping the level of perceived (Borg scale) and objectively measured exhaustion (heart rate) constant. Borg ratings will be averaged across all intervals of a given session (using the abbreviated Borg Scale 1-10). The following formula will be used to determine the starting training session (TS): ((Max performance [W] during spiroergometry / body weight [kg]) * 10) - 9. For example, a patient with a body weight of 80kg who reaches 240W in the spiroergometry will start training at: ((240 / 80) * 10) -9= 21, i.e. trainings session 21 (TS20). Each subsequent training session will increase duration as well as work load during each interval in a predefined manner (see Table 1).

**TABLE 1.** Selected training sessions (TS) from the training plan including an example of calculated performance and energy expended for a hypothetical patient with an anchor value of 60W (in red).

| *Training Session #* | *Interval #* | *Duration (min)* | *Intensity (% of anchor value)* | *Performance (W)* | *Energy expended* |
|---|---|---|---|---|---|
| **TS 10** | 1 | 5 | 100 | 60 | 300 |
| | 2 | 6 | 100 | 60 | 360 |
| | 3 | 6 | 100 | 60 | 360 |
| | 4 | 6 | 100 | 50 | 300 |
| | 5 | 4 | 100 | 50 | 200 |
| *Total* | | *27 min* | | | *91.200 Joule* |
| **TS 20** | 1 | 5 | 110 | 66 | 330 |
| | 2 | 10 | 120 | 72 | 720 |
| | 3 | 10 | 120 | 72 | 720 |
| | 4 | 10 | 110 | 66 | 660 |
| | 5 | 6 | 120 | 72 | 432 |
| *Total* | | *41 min* | | | *171.720 Joule* |
| **TS 30** | 1 | 8 | 110 | 66 | 528 |
| | 2 | 10 | 120 | 72 | 720 |
| | 3 | 15 | 120 | 72 | 1080 |
| | 4 | 10 | 120 | 72 | 720 |
| | 5 | 10 | 110 | 66 | 660 |
| | 6 | 8 | 110 | 66 | 528 |
| *Total* | | *61 min* | | | *254.160 Joule* |
| **TS 40** | 1 | 12 | 120 | 72 | 864 |
| | 2 | 15 | 130 | 78 | 1170 |
| | 3 | 15 | 150 | 90 | 1350 |
| | 4 | 15 | 140 | 84 | 1260 |
| | 5 | 12 | 120 | 72 | 864 |
| *Total* | | *69 min* | | | *330.480 Joule* |

*Advancing through the training plan:* Each interval of each training session (TS) will be Borg-rated (abbreviated Borg scale 1-10) and scores averaged across the TS. If the patient reaches an average Borg rating of 8 for the current session, the current level (e.g. TS10) will be repeated for the next session. Average Borg rating between 1 and 7 will result in advancing to a higher level. Average Borg ratings of 9 or 10 will result in decreasing the training intensity for the next session. If a patient is unable to complete all required intervals of a given session, he or she will drop back to the last fully completed TS in the training program. The algorithm for determining the next training session intensity is illustrated in Figure 2.

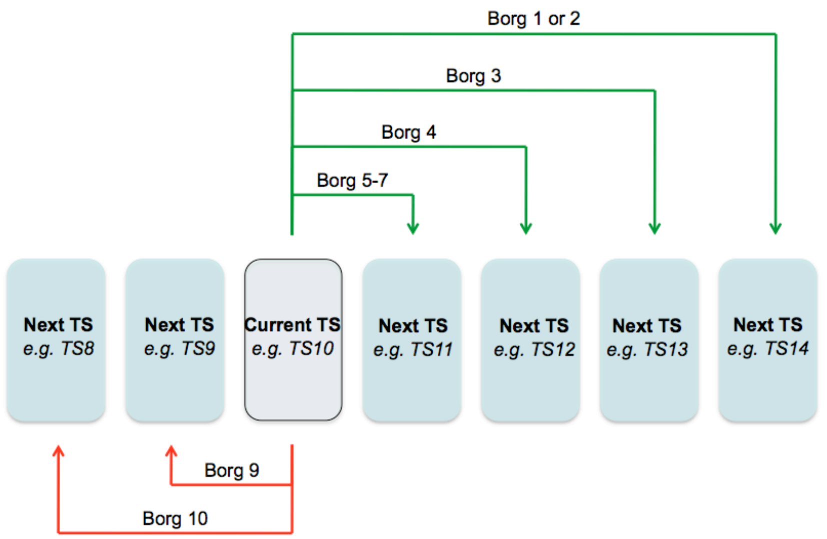


**Figure 2.** Algorithm for advancing through the training plan based on average Borg rating of the current training session (TS). A Borg rating of 8 will result in repeating the session.

The maximum length of any TS is 70 minutes (TS44). If a patient reaches TS44 (5 intervals: 10, 15, 15, 15 and 15 minutes with an intensity of 120, 140, 150, 150, and 150% power), they will repeat this level of intensity until the end of the 3 month training period.

Training sessions will be scheduled individually for each patient at the UKE Dept Physiotherapy and can be completed at the patient's convenience within the regular business hours Monday-Friday depending on the availability of training slots. During these times, experienced physiotherapists familiar with the AERCONN training program are available to supervise the training of the patients.

11 b: Explanation for choice of comparators:

The training will be compared to a wait-list control group which will receive the intervention after 6 months and a second ergometry was performed. We decided not to

use a sham-intervention because the effort of 2-3 sessions for three months is not to be justified based on ethical reasons for controls.

*11 c: Criteria for discontinuing or modifying allocated interventions for a given trial participant (e.g., drug dose change in response to harms, participant request, or improving/worsening disease).*

As a safety measure, spiroergometry will be stopped if a patient reaches systolic BP ≥ 200 and/or diastolic BP ≥ 110. If substantial ectopic electric heart activity or other complications occur, the spiroergometry will be stopped and the patient will be excluded from the study. Similarly, if any of these complications arise during the training program, the patient might be removed from the study due to safety concerns. This decision will be made after discussing the issue between the patient and the medical team (neurologist, exercise physiologist, physiotherapist).

Any patient might be excluded from the study when the training will be interrupted or was interrupted for more than 2 weeks or if training was reduced to one session/week for more than 2 weeks. Furthermore, patients experiencing persistent worsening of symptoms after training sessions lasting to the following day will be excluded.

*11 c: Strategies to improve adherence to intervention protocols, and any procedures for monitoring adherence (e.g., drug tablet return; laboratory tests).*

We will obtain detailed information about each patient’s time constraints and other commitments before inclusion to avoid drop-outs due to logistical difficulties. Particular attention will be given to potential problems with transfers to the study site and back. In addition, patients will be informed that symptoms might reappear or temporarily worsen during or soon after training sessions. Early telephone calls after 2-4 weeks of training will be used to detect barriers for adherence. During the last 2 weeks of training, a structured interview focusing on exercise motivation and barriers will be conducted with each participant. This interview will be repeated at month 6. Patients will also receive a summary feedback regarding their individual response to treatment with respect to physical fitness and clinical parameters.

*11d: Relevant concomitant care and interventions that are permitted or prohibited during the trial.*

In case of deterioration such as a relapse during the training program, the participant is requested to contact the study center and to discuss treatment option (e.g. steroid treatment) and / or training interruption. Steroid treatment of relapses and an interruption of the training of up to 2 weeks will be tolerated. Longer interruptions will be considered breach of protocol and the patient excluded from the per-protocol analysis (but retained in the intention-to-treat analysis).

**12 Outcomes:** *Primary, secondary, and other outcomes, including the specific measurement variable (e.g., systolic blood pressure), analysis metric (e.g., change from baseline, final value, time to event), method of aggregation (e.g., median, proportion), and time point for each outcome. Explanation of the clinical relevance of chosen efficacy and harm outcomes is strongly recommended.*

12.1. Endpoints

The primary, secondary, and tertiary endpoints of the trial are listed in Table 2 below and described in detail in the following paragraphs. In addition to these endpoints, several ancillary studies will be conducted to maximize the scientific knowledge gained regarding the neurobiological and physiological effects of exercise in patients with MS (see section 12.2.).

**Table 2.** Overview of primary, secondary, and tertiary endpoints of the AERCONN trial.

| Endpoint | Construct | Measure |
|---|---|---|
| **Primary endpoint** | Verbal Learning and Memory | VLMT |
| **Secondary endpoints** | Functional connectivity | rs MEG, rs fMRI |
| | Structural integrity and connectivity | GM density, DTI |
| | Other cognitive functions | Neuropsych. battery |
| **Tertiary endpoints** | Walking ability | 6MWT, accelerometry |
| | Motor coordination and balance | MFT-S3 |
| | Well-being and quality of life | Patient-reported outcomes |

12.1.1. Primary endpoint:

The primary endpoint of this study will be the Verbal Learning and Memory Scale (VLMT, Helmstaedter, Lendt and Lux, 2001) after 3 months of training. Learning and

memory are important domains of cognitive function and frequently impaired in MS, with important implications for quality of life, vocational status and psychosocial problems (Chiaravalotti and De Luca, 2008; Langdon, 2011). Moreover, MS patients rank memory and thinking amongst the most important body functions (Heesen et al., Mult Scler 2008).

Learning and memory is among the cognitive domains most consistently shown to improve after aerobic exercise training in numerous studies including patients with chronic diseases and aged healthy individuals (Smith et al., Psychosom Med 2010). Moreover, in our previous pilot trial of aerobic exercise in progressive MS (Briken et al., MSJ 2013), verbal learning and memory as measured by the VLMT was significantly increased after the training program.

12.1.2. Secondary endpoints:

Secondary endpoints include imaging-based measures of brain integrity and connectivity as well as neuropsychological tests covering several domains of higher-order brain function.

*Functional connectivity* will be assessed using resting-state MEG and fMRI analyses. It will be compared between patients and healthy controls both on sensor and source level. As measures we use quantities, which are robust to artifacts of volume conduction, specifically the imaginary part of coherency, power correlations corrected for mixing artifacts, and, on source level, multivariate measures, which optimize source orientation to detect neural interactions. Although the approach is exploratory we will specifically concentrate on motor systems and working memory and relate results to findings from structural connectivity analysis.

*Structural connectivity* will be evaluated by measuring the fractional anisotropy (FA) calculated from Diffusion-Tensor Imaging data (DTI). Overall cortical es well as regional (i.e. corpus callosum, primary and secondary motor cortex) FA values will be obtained. In addition, connectivity will be analyzed by probabilistic tractography focusing on the motor system as well as brain areas involved in processing speed and working memory as the core neuropsychological deficit in MS and correlated with neuropsycological parameters as well as functional connectivity measures.

*Structural integrity of gray matter* will be assessed by gray matter density measurement on T1 weighted MR images. Here, we will obtain overall and focal (cortex, hippocampus, thalamus) gray matter density measures

In addition to imaging makers of brain structure and connectivity, we will analyze several domains of *higher-order brain function* as secondary endpoints. These include attention (Test of Attentional Performance, TAP), visuospatial learning and memory (Brief Visuospatial Memory Test Revised, BVMT-R, Benedict et al. 1997), processing speed (Symbol Digit Modalities Test, SDMT, Smith 1973, Paced Auditory Serial Addition Task, PASAT, Gronwall, 1977), working memory (Corsi Block, Corsi 1972), and social cognition (theory of mind as determined by the Movie for Assessment of Social Cognition, Dziobek et al., 2006). Cognitive function is of major clinical importance in MS since is linked to quality of life and vocational status. In addition, currently available disease-modifying therapies have limited effects on cognition and attempts to develop pharmacological therapies for cognitive impairment have been unsuccessful in MS (Benedict & Zivadinov Nat Rev Neurol 2011). Therefore, exploring novel treatment options for cognitive dysfunction in MS such as exercise is highly relevant for MS care.

12.1.3. Tertiary endpoints:

Tertiary outcomes include objective measures of *walking ability*. We will obtain total distance (in meters) during the six-minute walk test (6MWT) as well as “real-life” walking speed as measured by the Actibelt® over the course of 7 days.

Furthermore, we will obtain patient-reported outcome measures (PRO) to assess subjective well-being and quality of life. These measures include the Inventory of Depressive Symptoms (IDS-30SR), the Fatigue Scale for Motor and Cognitive Function (FSMC, Penner et al., 2009), the Hamburg Quality of Life Questionnaire for Multiple Sclerosis (HAQUAMS, Gold et al., 2001), and the Multiple Sclerosis Walking Scale-12 (MSWS-12, Hobart et al.)

Finally, we will objectively assess motor coordination and balance using the MFT-S3.

12.2. Ancillary studies:

In order to maximize the scientific insight that can be gained from this phase IIa trial with respect to the neurobiology and physiology of exercise in multiple sclerosis, we will conduct a series of ancillary studies that address potential biomarkers, changes in CNS pathology, aerobic fitness and metabolic effects on fat and muscle tissue as well as psychological factors that might influence treatment response and adherence.

To explore effects of exercise on MS-related gray and white matter pathology, we will obtain comprehensive MRI-based assessments of lesion load and pattern (double

inversion recovery (DIR), T2w, fluid-attenuated-inversion revovery (FLAIR), T1w), global and regional alterations of normal appearing gray and white matter (magnetization transfer (MTR), T2 prime (T2’), arterial spin labeling (ASL)), global and regional atrophy (e.g . brain parenchymal fraction, hippocampal subregional volumes, cortical thickness). Moreover, we will explore exercise effects on functional coupling during a cognitive task using task MEG (PASAT) and task fMRI (n-back with increasing complexity (2-back, 3-back, 4-back)).

Several myokines and cytokines have been hypothesized to mediate the beneficial effects of exercise on metabolic function as well as the CNS. We will use the opportunity of the AERCONN trial to study potential biomarkers such as brain-derived neurotrophic factor (BDNF) and the recently discovered myokine irisin in serum and plasma and investigate their association with clinical and imaging-based measures of brain function and integrity. New evidence from animal models suggests that the irisin pathway may be a key regulator involved in fat “browning” and other metabolic functions (Boström Nature 2012). Furthermore, irisin may directly induce “neuroprotective” molecules such as BDNF in the hippocampus (Wrann Cell Metab 2013) and could therefore be an interesting peripheral biomarker for CNS effects of exercise. Therefore, we will use bioimpedance measurements to monitor fat and muscle composition as well as our clinical and neuroimaging-based measures of brain structure and function to explore if these associations can also be seen in humans.

Lastly, we will obtain standardized questionnaires covering training motivation and maintenance at month 3 and month 6 to explore psychological factors that may facilitate or impede treatment adherence.

**13 Participant timeline (see Figure 3):** *Time schedule of enrolment, interventions (including any run-ins and washouts), assessments, and visits for participants. A schematic diagram is highly recommended.*

Interested patients will first be contacted by telephone to screen eligibility. Eligible patients will then be enrolled in the study and baseline assessment appointments will be scheduled. All outcome parameters will be assessed at month 0 (baseline) and month 3 (post intervention). *Each of these assessment blocks* includes 5 separate days of assessment (see Figure 3): *Day 1*: Visit to the MS outpatient center for neurological scoring, neuropsychology etc; *Day 2*: Visit to the Dept Neurophysiology for MEG. *Day 3* and Day 4: Visit to the Dept Neuroradiology for MRI. *Day 5*: Visit to the Dept Sports

Medicine for spiroergometry etc. During the entire assessment block (approx 7 days), the patients will wear the Actibelt® accelerometer. Randomization will be performed AFTER completion of day 5 in assessment block Wk 0.

Day 1 Assessment plan: During the clinical visit to the MS Center of the UKE, the following steps will be completed: Informed consent, blood sample (10 min), demographics and EDSS (10 min), patient self-report questionnaires (IDS-30SR, HAQUAMS, FSMC, MSWS-12, 30 min), neuro-psychological testing battery (90 min), 9-HPT (5 min), 6MWT and T25 (10 min), MASC (20 min).

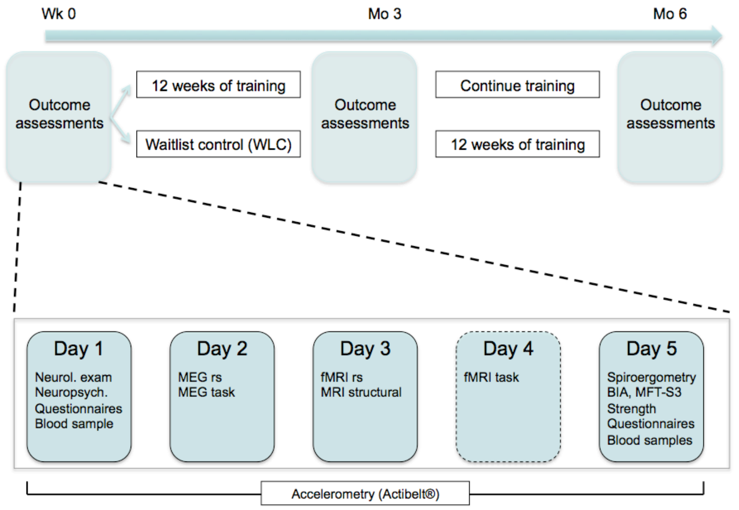


**Figure 3.** Participant timeline with all required assessments at baseline (month 0), post intervention (month 3), and follow-up (month 6). Each assessment block consists of 5 assessment days (see inset).

Day 2: MEG assessments.

Day 3: Structural MRI assessments.

Day 4: Functional MRI assessments. Day 4 will only be done in 1/3 of the participants (i.e. the first 20 patients who enter the trial).

Day 5 Assessment plan: The following assessments are conducted at the Dept Sports Medicine (in this order): Height and weight measurement (3 min), resting ECG (4 min), Bioelectrical Impedance Analysis (BIA, 6 min), spiroergometry (including blood draws before and after, 5ml plasma EDTA each, approx. 35 min), MFT-S3 assessment of balance and trunk stability (12 min), strength assessment (35 min), additional questionnaires (IPAQ (Lee 2011), Stadienzugehörigkeit im Transtheoretischen Modell (Basler 1999), Intention und Sportbezogene Selbstkonkordanz (Seelig & Fuchs 2006) , Sportbezogene Konsequenzerwartung (Fuchs 1994), Sportbezogene Situative Barrieren und Management (Krämer & Fuchs 2010), sportbezogene sozialen Unterstützung (Fuchs 2008), Sportbezogene Selbstwirksamkeit (approx. 30 min)).

**14 Sample size:** *Estimated number of participants needed to achieve study objectives and how it was determined, including clinical and statistical assumptions supporting any sample size calculations*

Sample size was calculated based on results obtained in the pilot study on exercise and cognitive function in progressive MS (Briken et al., MSJ 2013). Below are the power calculations for the VLMT learning (trials 1 through 5) and delayed memory (trial 7).

<u>14.1. VLMT Learning:</u>

*Numeric Results for Two-Sample T-Test*

Null Hypothesis: Mean1=Mean2. Alternative Hypothesis: Mean1<>Mean2

The standard deviations were assumed to be unknown and equal.

| **Power** | **N1** | **N2** | **Ratio** | **Alpha** | **Beta** | **Mean1** | **Mean2** | **S1** | **S2** |
|---|---|---|---|---|---|---|---|---|---|
| 0.90388 | 20 | 20 | 1.000 | 0.02500 | 0.09612 | 4.0 | 9.8 | 5.0 | 5.0 |
| 0.81609 | 16 | 16 | 1.000 | 0.02500 | 0.18391 | 4.0 | 9.8 | 5.0 | 5.0 |
| 0.90639 | 17 | 17 | 1.000 | 0.05000 | 0.09361 | 4.0 | 9.8 | 5.0 | 5.0 |
| 0.80985 | 13 | 13 | 1.000 | 0.05000 | 0.19015 | 4.0 | 9.8 | 5.0 | 5.0 |

Group sample sizes of 20 and 20 achieve 90% power to detect a difference of -5.8 between the null hypothesis that both group means are 4.0 and the alternative hypothesis that the mean of group 2 is 9.8 with estimated group standard deviations of 5.0 and 5.0 and with a significance level (alpha) of 0.02500 using a two-sided two-sample t-test.

<u>14.2. VLMT Delayed recall:</u>

*Numeric Results for Two-Sample T-Test*

Null Hypothesis: Mean1=Mean2. Alternative Hypothesis: Mean1<>Mean2

The standard deviations were assumed to be unknown and equal.

| **Power** | **N1** | **N2** | **Ratio** | **Alpha** | **Beta** | **Mean1** | **Mean2** | **S1** | **S2** |
|---|---|---|---|---|---|---|---|---|---|
| 0.91886 | 17 | 17 | 1.000 | 0.02500 | 0.08114 | 0.6 | 3.2 | 2.0 | 2.0 |
| 0.81514 | 13 | 13 | 1.000 | 0.02500 | 0.18486 | 0.6 | 3.2 | 2.0 | 2.0 |
| 0.91148 | 14 | 14 | 1.000 | 0.05000 | 0.08852 | 0.6 | 3.2 | 2.0 | 2.0 |
| 0.82630 | 11 | 11 | 1.000 | 0.05000 | 0.17370 | 0.6 | 3.2 | 2.0 | 2.0 |

Group sample sizes of 17 and 17 achieve 92% power to detect a difference of -2.6 between the null hypothesis that both group means are 0.6 and the alternative hypothesis that the mean of group 2 is 3.2 with estimated group standard deviations of 2.0 and 2.0 and with a significance level (alpha) of 0.02500 using a two-sided two-sample t-test.

<u>14.3. Summary statement:</u>

Based on these calculations, powering for the dual primary endpoints VLMT learning and VLMT delayed recall, 90% power will be achieved with n=20 patients in each

arm of the trial. To safeguard against dropouts (based on previous experience, drop-out in behavioral trials often reach 20%) and the possibility that effect sizes in the pilot trial may have overestimated the real effect, we will enrol n=60 patients in the trial so that we will have approximately n=30 in each trial arm.

**15 Recruitment:** *Strategies for achieving adequate participant enrolment to reach target sample size.*

The Hamburg MS center has extensive expertise in patient recruitments for various behavioral and pharmacological therapeutic interventions in MS including 4 investigator-initiated phase II drug trials, 2 phase I/IIa exercise studies, and several phase II behavioral intervention trials including RCTs of patient education programs and a CBT-based online intervention.

We will screen the registry database of the MS Day Hospital (n>7500) for patients who meet inclusion criteria (see below) and have indicated an interest in information about new clinical studies. These patients will be contacted by mail and invited to apply for participation in the trial. In addition advertisements for the study will be posted via the local self help society as well as the local MS network (www.ms-netz-hamburg.de).

Based on the recruitment in a previous exercise trial with more disabled patients and recent experiences within the NEUCONN-1 project, we expect to recruit and enroll 60 patients within 12 months.

**16 Allocation**

*16a Sequence generation: Method of generating the allocation sequence (e.g., computer-generated random numbers), and list of any factors for stratification. To reduce predictability of a random sequence, details of any planned restriction (e.g., blocking) should be provided in a separate document that is unavailable to those who enrol participants or assign interventions.*

Subjects will be randomized 1:1 to the exercise group or waitlist control using a computer-based random numbers generator. The randomization procedure does not include any stratification factors and no other restrictions (such as blocking).

*16b Concealment mechanism: Mechanism of implementing the allocation sequence (e.g., central telephone; sequentially numbered, opaque, sealed envelopes), describing any steps to conceal the sequence until interventions are assigned.*

A sequence of random numbers (1 or 2) will be generated by Eik Vettorazzi, placed in a sealed envelop, and stored a locked drawer in the office of the Dept Medical Biometry

and Epidemiology of the UKE. Every time a patient has completed day 5 of the Month 0 assessment (concealed allocation), Anja Fischer will call the secretary and obtain allocation for this subject. The entire random number sequence will never be accessible to any researcher involved in data acquisition, analysis, interpretation, and conductance of the AERCONN trial.

*16c Implementation: Who will generate the allocation sequence, who will enrol participants, and who will assign participants to interventions.*

The allocation sequence will be generated by Eik Vettorazzi (EV) and kept sealed by the secretary of the Dept Medical Biometry and Epidemiology.

**17: Blinding**

*17a Blinding: Who will be blinded after assignment to interventions (e.g., trial participants, care providers, outcome assessors, data analysts), and how.*

This is a single-blind, randomized, controlled trial. Blinding of participants is not possible. However, all outcome measures will be obtained by assessors blind to group assignment of the patient and patients will be instructed not to disclose any information during the assessments that may reveal their group assignment to the assessor. Debriefing of the patients will be conducted by staff not involved in obtaining outcome measures.

All data processing (scoring of neurological and neuropsychological tests, image analyses of MRI and MEG data, spirometry data etc.) will be analyzed blind to patient identity and group assignment.

Breaking of the study code and statistical analyses will be performed after completion of all data assessments and conducted by a biostatistician (EV) not involved in data acquisition and data preparation.

*17b Emergency unblinding: If blinded, circumstances under which unblinding is permissible, and procedure for revealing a participant's allocated intervention during the trial.*

Not applicable since patients and physiotherapist will know whether or not the patient is currently on treatment and initiate all necessary safety precautions without breaking the study code.

**18: Data collection methods**

*18a: Plans for assessment and collection of outcome, baseline, and other trial data, including any related processes to promote data quality (e.g., duplicate measurements,*

*training of assessors) and a description of study instruments (e.g., questionnaires, laboratory tests) along with their reliability and validity, if known. Reference to where data collection forms can be found, if not in the protocol.*

Management of screening, inclusion and study conduct will be based on the internet-based project-management tool attached to the HAPIMS database. This database uses secure data management and has been approved for use by the responsible Data Protection and Security Office of the UKE. Data will be collected on paper-based source documents (neuropsychological assessments, performance-based measures, questionnaires) or primary computer-based (MRI, MEG) or chip-based (accelerometry). Data will be uploaded on a protected web-based project-platform to allow an integrated view on all assessments.

Assessment of safety: DSMB reports will be prepared biannually. As we do not expect adverse events DSMB recommendations for taking patients out or stopping the study seem highly unlikely.

*18b Retention: Plans to promote participant retention and complete follow-up, including list of any outcome data to be collected for participants who discontinue or deviate from intervention protocols.*

See 11c.

**19 Data management:** *Plans for data entry, coding, security, and storage, including any related processes to promote data quality (e.g., double data entry; range checks for data values). Reference to where details of data management procedures can be found, if not in the protocol.*

Data-input from source documents will be double-checked by 2 persons.

**20 Statistical methods**

*20a: Statistical methods for analyzing primary and secondary outcomes. Reference to where other details of the statistical analysis plan can be found, if not in the protocol.*

The primary analyses will compare changes from Month 0 to Month 3 between the exercise group and the waitlist control group. According to guidelines for statistical analysis of clinical trials published by The European Agency for the Evaluation of Medicinal Products (CPMP/ICH/363/96 and CPMP/EWP/2863/99), we will computed the primary statistical analysis for all outcomes using ANCOVA models adjusting for baseline measurements of the respective outcome variable to evaluate treatment effects (measured

as change from baseline). No other covariates will be included in this primary analysis. As recommended, this model will also not include treatment by covariate interactions.

All primary analyses will be conducted as intention-to-treat (ITT) including all patients who have received group allocation. Every effort will be made to obtain Month 3 and Month 6 data from all participants (even if they drop out of the exercise program). However, in case of missing data, primary ITT analyses will be conducted using a Last-Observation-Carried-Forward (LOCF) approach.

As sensitivity analyses, we will compute the same ANCOVA models as per-protocol (i.e. including only patients who completed the 12 weeks of training with at least 2 sessions in each week) as well as non-parametric intent-to-treat (ITT) analyses using Kruskal-Wallis tests where patients who drop out are assigned the lowest rank.

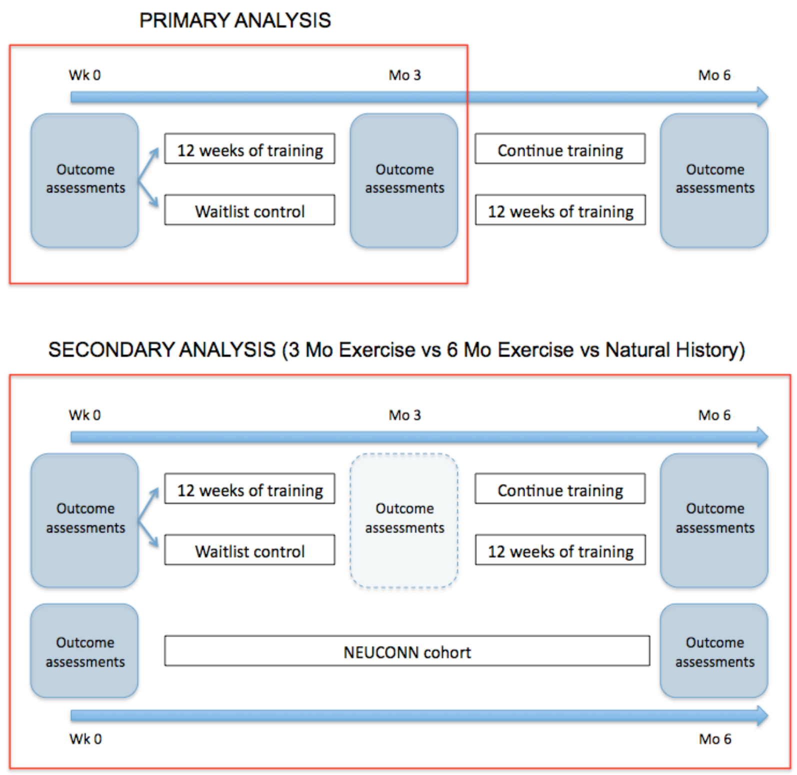


**Figure 4.** Analysis plan

*20c: Definition of analysis population relating to protocol nonadherence (e.g., as-randomized analysis), and any statistical methods to handle missing data (e.g., multiple imputation)*

Data analysis will be ITT as well as PP. For ITT analyses, missing data will be handled using a Last-Observation-Carried-Forward (LOCF) approach.

**21: Monitoring**

*21a: Composition of DMC; summary of its role and reporting structure; statement of whether it is independent from the sponsor and competing interests; and reference to where further details about its charter can be found, if not in the protocol. Alternatively, an explanation of why a DMC is not needed.*

Monitoring will be through biannual reporting to the Neu2 Steering committee.

*21b: Description of any interim analyses and stopping guidelines, including who will have access to these interim results and make the final decision to terminate the trial*

Not planned.

**22 Harms:** *Plans for collecting, assessing, reporting, and managing solicited and spontaneously reported adverse events and other unintended effects of trial interventions or trial conduct*

Based on the literature as well as own study experiences especially in progressive MS patients, we do not expect adverse events: However, some patients might experience temporary worsening of symptoms which might lead to adjustment of the training.

**23 Auditing**: *Frequency and procedures for auditing trial conduct, if any, and whether the process will be independent from investigators and the sponsor*

There are no in advance planned audits. However, regulatory authorities might choose to audit the study.

## IV) Ethics and dissemination

**26 Research ethics approval:** *Plans for seeking REC/IRB approval*

Ethical approval has been obtained from the ethical committee of Hamburg chamber of physicians (EthikNr. PV4356, approval granted March 15, 2013).

**27 Protocol amendments:** *Plans for communicating important protocol modifications (e.g., changes to eligibility criteria, outcomes, analyses) to relevant parties (e.g., investigators, RECs/IRBs, trial participants, trial registries, journals, regulators)*

The DMSB will need to approve major changes of the study protocol e.g. concerning outcome measures. Also an amendment of the study protocol would be submitted to the ethical committees. Minor changes to the protocol, which will not affect the conduction of the study, will be communicated to the DSMB. Information about changes will be added to the study registration.

**28 Consent or assent**

*28a: Who will obtain informed consent or assent from potential trial participants or authorized surrogates, and how (see item 32)*

Consent will be obtained through neurologists working within the MS day hospital.

*28b Additional consent provisions for collection and use of participant data and biological specimens in ancillary studies, if applicable.*

Usage of biological samples (PBMCs, plasma, serum) for ancilliary studies are covered by the AERCONN patient consent form.

**29 Confidentiality:** *How personal information about potential and enrolled participants will be collected, shared, and maintained in order to protect confidentiality before, during, and after the trial*

All personal information will be entered into the database by members of the research team. Data will be anonymized and there will be no possibility to link data back to individual patients.

**30 Declaration of interests:** *Financial and other competing interests for principal investigators for the overall trial and each study site.*

CH has received research grants, congress travel compensations as well as honoraria for presentations from BiogenIdec, Genzyme, Sanofi-Aventis, Bayer Healthcare, Merck Serono, Teva Aventis, and Novartis.

SMG has received honoraria for consultancy and presentations from Novartis and congress travel compensations from BiogenIdec, Merck Serono, and Teva Aventis.

IKP has received research grants from Bayer Switzerland AG as well as honoraria for serving as speaker at scientific meetings, consultant, and as member of scientific advisory board for Actelion, Bayer Pharma AG, Biogen Idec, Merck Serono, Novartis, and Teva Aventis.

KHS has received research grants from Hexal Medical.

AKE and GN have no competing interests.

**31 Access to data:** *Statement of who will have access to the final trial data set, and disclosure of contractual agreements that limit such access for investigators*

The study center will coordinate the intra-study data sharing process. All Principal Investigators will be given access to the cleaned data sets.

**32 Ancillary and post-trial care:** *Provisions, if any, for ancillary and post-trial care, and for compensation to those who suffer harm from trial participation*

Not applicable.

### 33) Dissemination policy

*33a: Plans for investigators and sponsor to communicate trial results to s, health care professionals, the public, and other relevant groups (e.g., via publication, reporting in results databases, or other data-sharing arrangements), including any publication restrictions.*

Results will be published in major journals and presented at scientific conferences (European Committee for Treatment and Research in Multiple Sclerosis (ECTRIMS), Rehabilitation in Multiple Sclerosis (RIMS). Furthermore, it is planned to publish main results on relevant patient websites. All patients will receive structured feedback and copies of major publications.

If the trial is successful a larger phase-3 study will be planned.

*33b: Authorship eligibility guidelines and any intended use of professional writers*

Authorship will be shared between persons involved in the study following the current guidelines of the International Committee of Medical Journal Editors (ICMJE). No professional writers or persons not directly involved in the study will be granted authorship.

### 34) References

Barkhof F, Calabresi PA, Miller DH, Reingold SC (2009) Imaging outcomes for neuroprotection and repair in multiple sclerosis trials. Nat Rev Neurol 5: 256-266

Bermel RA, Bakshi R (2006) The measurement and clinical relevance of brain atrophy in multiple sclerosis. Lancet Neurol 5: 158-170

Bielekova B, Martin R (2004) Development of biomarkers in multiple sclerosis. Brain 127: 1463-1478

Bonavita S, Gallo A, Sacco R, Corte MD, Bisecco A, Docimo R, Lavorgna L, Corbo D, Costanzo AD, Tortora F, Cirillo M, Esposito F, Tedeschi G (2011) Distributed changes in default-mode resting-state connectivity in multiple sclerosis. Mult Scler 17: 411-422

Boström P, Wu J, Jedrychowski MP, Korde A, Ye L, Lo JC, Rasbach KA, Boström EA, Choi JH, Long JZ, Kajimura S, Zingaretti MC, Vind BF, Tu H, Cinti S, Højlund K, Gygi SP, Spiegelman BM (2012) A PGC1-a-dependent myokine that drives brown-fat-like development of white fat and thermogenesis. Nature 481: 463-468

Briken S, Gold SM, Ketels G, Patra S, Daumer M, Schulz KH, Heesen C (2013) Comparison of different endurance trainings in advanced multiple sclerosis patients: A randomized - controlled pilot study. Mult Scler J (in press)

Buckner RL, Vincent JL (2007) Unrest at rest: default activity and spontaneous network correlations. Neuroimage 37: 1091-1096

Cotman CW, Berchtold NC, Christie LA (2007) Exercise builds brain health: key roles of growth factor cascades and inflammation. Trends Neurosci 30: 464-472

Cotman, CW, Berchtold, NC (2002). Exercise: a behavioral intervention to enhance brain health and plasticity. Trends Neurosci. 25: 295–301.

Cover KS, Vrenken H, Geurts JJ, van Oosten BW, Jelles B, Polman CH, Stam CJ, van Dijk BW (2006) Multiple sclerosis patients show a highly significant decrease in alpha band interhemispheric synchronization measured using MEG. Neuroimage 29: 783-788

Donner TH, Siegel M, Fries P, Engel AK (2009) Build-up of choice-predictive activity in human motor cortex during perceptual decision making. Curr Biol 19: 1581-1585

Engel AK, Fries P, Singer W (2001) Dynamic predictions: oscillations and synchrony in top-down processing. Nat Rev Neurosci 2: 704-716

Etnier JL, Nowell PM, Landers DM, Sibley BA (2006) A meta-regression to examine the relationship between aerobic fitness and cognitive performance. Brain Res Rev 52: 119-130

Faivre A, Rico A, Zaaraoui W, Crespy L, Reuter F, Wybrecht D, Soulier E, Malikova I, Confort-Gouny S, Cozzone PJ, Pelletier J, Ranjeva JP, Audoin B (2012) Assessing brain connectivity at rest is clinically relevant in early multiple sclerosis. Mult Scler. DOI: 10.1177/1352458511435930

Filippi M, Rocca MA (2011) The role of magnetic resonance imaging in the study of multiple sclerosis: diagnosis, prognosis and understanding disease pathophysiology. Acta Neurol Belg 111: 89-98

Fischer JS, Priore RL, Jacobs LD, Cookfair DL, Rudick RA, Herndon RM (2000) Neuropsychological effects of interferon beta-1a in relapsing multiple sclerosis. Ann Neurol 48: 885–892

Fox MD, Snyder AZ, Vincent JL, Corbetta M, Van Essen DC, Raichle ME (2005) The human brain is intrinsically organized into dynamic, anticorrelated functional networks. Proc Natl Acad Sci U S A 102: 9673-9678

Gleeson M, Bishop NC, Stensel DJ, Lindley MR, Mastana SS, Nimmo MA (2011) The anti-inflammatory effects of exercise: mechanisms and implications for the prevention and treatment of disease. Nat Rev Immunol 2011 11: 607-615

Gold SM, Schulz KH, Hartmann S, Mladek M, Lang UE, Hellweg R, Reer R, Braumann KM, Heesen C (2003). Basal serum levels and reactivity of nerve growth factor (NGF) and brain-derived neurotrophic factor (BDNF) to standardized acute exercise in multiple sclerosis and controls. J Neuroimmunol 138, 99-105.

Gold SM, O'Connor MF, Gill R, Kern KC, Shi Y, Henry RG, Pelletier D, Mohr DC, Sicotte NL (in press). Detection of altered hippocampal morphology in multiple sclerosis-associated depression using automated surface mesh modeling. Hum Brain Mapp.

Gold SM, Kern KC, O'Connor MF, Montag M, Kim A, Yoo YS, Giesser BS, Sicotte NL (2010). Smaller cornu ammonis 2-3 / dentate gyrus volumes and elevated cortisol in multiple sclerosis patients with depressive symptoms. Biol Psychiatry 68(6):553-9.

Hagiwara K, Okamoto T, Shigeto H, Ogata K, Somehara Y, Matsushita T, Kira J, Tobimatsu S (2010) Oscillatory gamma synchronization binds the primary and secondary somatosensory areas in humans. Neuroimage 51: 412-420

Hawellek DJ, Hipp JF, Lewis CM, Corbetta M, Engel AK (2011) Increased functional connectivity indicates the severity of cognitive impairment in multiple sclerosis. Proc Natl Acad Sci USA 108: 19066-19071

Hipp JF, Engel AK, Siegel M (2011) Oscillatory synchronization in large-scale cortical networks predicts perception. Neuron 69: 387-396

Hipp JF, Hawellek D, Corbetta M, Siegel M, Engel AK (2012) The large-scale organization of spontaneous oscillatory neuronal activity. Nat Neurosci 15: 884-890

Heesen C, Romberg A, Gold S, Schulz KH (2006) Physical exercise in multiple sclerosis: supportive care or a putative disease-modifying treatment. Expert Rev Neurother 6: 347-355

Heesen C, Schulz KH, Fiehler J, Von der Mark U, Otte C, Jung R, Poettgen J, Krieger T, Gold SM (2010). Correlates of cognitive dysfunction in multiple sclerosis. Brain Behav Immunity 24(7):1148-55.

Hillman CH, Erickson KI, Kramer AF (2008) Be smart, exercise your heart: exercise effects on brain and cognition. Nat Rev Neurosci 9: 58-65

Kiy G, Lehmann P, Hahn HK, Eling P, Kastrup A, Hildebrandt H (2011). Decreased hippocampal volume, indirectly measured, is associated with depressive symptoms and consolidation deficits in multiple sclerosis. Mult Scler 17(9):1088-97.

Le Page C, Bourdoulous S, Béraud E, Couraud PO, Rieu M, Ferry A (1996) Effect of physical exercise on adoptive experimental auto-immune encephalomyelitis in rats. Eur J Appl Physiol Occup Physiol 73: 130-135

Lowe MJ, Beall EB, Sakaie KE, Koenig KA, Stone L, Marrie RA, Phillips MD (2008) Resting state sensorimotor functional connectivity in multiple sclerosis inversely correlates with transcallosal motor pathway transverse diffusivity. Hum Brain Mapp 29: 818-827

Marzetti L, Del Gratta C, Nolte G (2008) Understanding brain connectivity from EEG data by identifying systems composed of interacting sources. Neuroimage 42: 87-98

Mullard A (2011) Success of immunomodulators in MS shifts discovery focus to neuroprotection. Nat Rev Drug Discov 10: 885-887

Nolte G, Marzetti L, Valdes Sosa P (2009) Minimum Overlap Component Analysis (MOCA) of EEG/MEG data for more than two sources. J Neurosci Meth 183: 72-76

Oken BS, Kishiyama S, Zajdel D, Bourdette D, Carlsen J, Haas M, Hugos C, Kraemer DF, Lawrence J, Mass M (2004) Randomized controlled trial of yoga and exercise in multiple sclerosis. Neurology 62: 2058-2064

Pedersen BK (2011) Exercise-induced myokines and their role in chronic diseases. Brain Behav Immun 25: 811-816

Penner IK, Kappos L, Rausch M, Opwis K, Radü EW (2006) Therapy-induced plasticity of cognitive functions in MS patients: insights from fMRI. J Physiol Paris 99: 455-462

Prakash RS, Patterson B, Janssen A, Abduljalil A, Boster A (2011) Physical activity associated with increased resting-state functional connectivity in multiple sclerosis. J Int Neuropsychol Soc 17: 986-997

Prakash RS, Snook EM, Motl RW, Kramer AF (2010) Aerobic fitness is associated with gray matter volume and white matter integrity in multiple sclerosis. Brain Res 1341: 41-51

Rossi S, Furlan R, De Chiara V, Musella A, Lo Giudice T, Mataluni G, Cavasinni F, Cantarella C, Bernardi G, Muzio L, Martorana A, Martino G, Centonze D (2009) Exercise attenuates the clinical, synaptic and dendritic abnormalities of experimental autoimmune encephalomyelitis. Neurobiol Dis 36: 51-59

Raichle ME (2010) Two views of brain function. Trends Cogn Sci 14: 180-190

Raichle ME, Snyder AZ (2007) A default mode of brain function: a brief history of an evolving idea. Neuroimage 37: 1083-1090

Rocca MA, Valsasina P, Absinta M, Riccitelli G, Rodegher ME, Misci P, Rossi P, Falini A, Comi G, Filippi M (2010) Default-mode network dysfunction and cognitive impairment in progressive MS. Neurology 74: 1252-1259

Roosendaal SD, Schoonheim MM, Hulst HE, Sanz-Arigita EJ, Smith SM, Geurts JJ, Barkhof F (2010) Resting state networks change in clinically isolated syndrome. Brain 133: 1612-1621

Schoonheim MM, Geurts JJ, Landi D, Douw L, van der Meer ML, Vrenken H, Polman CH, Barkhof F, Stam CJ (2011) Functional connectivity changes in multiple sclerosis patients: A graph analytical study of MEG resting state data. Hum Brain Mapp. DOI: 10.1002/hbm.21424

Schulz KH, Gold SM, Witte J, Bartsch K, Lang UE, Hellweg R, Reer R, Braumann MK, Heesen C (2004). Impact of aerobic training on immune-endocrine parameters, neurotrophic factors, quality of life and coordinative function in multiple sclerosis. J Neurol Sci, 225(1-2), 11-18.

Schulz KH, Meyer A, Langguth L (2012) Körperliche Aktivität und psychische Gesundheit. Bundesgesundheitsblatt 55: 55-65

Senkowski D, Schneider TR, Foxe JJ, Engel AK (2008) Crossmodal binding through neural coherence: implications for multisensory processing. Trends Neurosci 31: 401-409

Senkowski D, Kautz J, Hauck M, Zimmermann R, Engel AK (2011) Emotional facial expressions modulate pain-induced beta and gamma oscillations in sensorimotor cortex. J Neurosci 31: 14542-14550

Sicotte NL, Kern KC, Giesser BS, Arshanapalli A, Schultz A, Montag M, Wang H, Bookheimer SY (2008). Regional hippocampal atrophy in multiple sclerosis. Brain 131(Pt 4):1134-41.

Siegel M, Donner TH, Oostenveld R, Fries P, Engel AK (2007) High-frequency activity in human visual cortex is modulated by visual motion strength. Cereb Cortex 17: 732-741

Siegel M, Donner TH, Oostenveld R, Fries P, Engel AK (2008) Neuronal synchronization along the dorsal visual pathway reflects the focus of spatial attention. Neuron 60: 709-719

Siegel M, Donner TH, Engel AK (2012) Spectral fingerprints of large-scale neuronal interactions. Nat Rev Neurosci 13: 121-134

Smith PJ, Blumenthal JA, Hoffman BM, Cooper H, Strauman TA, Welsh-Bohmer K, Browndyke JN, Sherwood A (2010) Aerobic exercise and neurocognitive performance: a meta-analytic review of randomized controlled trials. Psychosom Med 72: 239-252

Staffen W, Mair A, Zauner H, Unterrainer J, Niederhofer H, Kutzelnigg A, Ritter S, Golaszewski S, Iglseder B, Ladurner G (2002) Cognitive function and fMRI in patients with multiple sclerosis: evidence for compensatory cortical activation during an attention task. Brain 125: 1275-1282

Tecchio F, Zito G, Zappasodi F, Dell' Acqua ML, Landi D, Nardo D, Lupoi D, Rossini PM, Filippi MM (2008) Intra-cortical connectivity in multiple sclerosis: a neurophysiological approach. Brain 131: 1783-1792

van den Elskamp IJ, Boden B, Dattola V, Knol DL, Filippi M, Kappos L, Fazekas F, Wagner K, Pohl C, Sandbrink R, Polman CH, Uitdehaag BM, Barkhof F (2010) Cerebral atrophy as outcome measure in short-term phase 2 clinical trials in multiple sclerosis. Neuroradiology 52: 875-881

Valsasina P, Rocca MA, Absinta M, Sormani MP, Mancini L, De Stefano N, Rovira A, Gass A, Enzinger C, Barkhof F, Wegner C, Matthews PM, Filippi M (2011) A multicentre study of motor functional connectivity changes in patients with multiple sclerosis. Eur J Neurosci 33: 1256-1263

Womelsdorf T, Schoffelen J-M, Oostenveld R, Singer W, Desimone R, Engel AK, Fries P (2007) Modulation of neuronal interactions through neuronal synchronization. Science 316: 1609-1612
