# Supplementary material for: Short-term interval aerobic exercise training does not improve memory functioning in relapsing-remitting multiple sclerosis—a randomized controlled trial
Source: PeerJ. 2018 Dec 12;6:e6037. doi: 10.7717/peerj.6037 (PMC6295157; doi:10.7717/peerj.6037)
Supplement: Supplemental Information 4 — mean values of raw scores (standard deviation). IG: Intervention group; CG: Control group. VLMT: Verbal Learning and Memory Test; SDMT: Symbol Digit Modalities Test; BVMT-R: Brief Visuospatial Memory Test-Revised; TAP Tonic Alertness: Test Battery for Attention Tonic Alertness; TAP Phasic Alertness: Test Battery for Attention Phasic Alertness; TAP CSA valid: Test Battery for Attention covert shift of attention valid; TAP CSA invalid: Test Battery for Attention covert shift of attention invalid; TAP Incompatibility: Test Battery for Attention Incompatibility; RWT: Regensburger Verbal Fluency Test; PASAT: Paced Auditory Serial Addition Test; MASC: Movie for Assessment of Social Cognition; *ANCOVA. [file peerj-06-6037-s004.docx]

|  | IG_PP | | | | CG | | | |  | Mean between group-difference  [95% CI] | | f-value* | p-value* | Effect-size*  Partial eta sq |  |
| --- | --- | --- | --- | --- | --- | --- | --- | --- | --- | --- | --- | --- | --- | --- | --- |
|  | Baseline | | Week 12 | | Baseline | | Week 12 | |  |  |  |  |  |  |  |
|  | n = 23 | | n = 23 | | n = 34 | | n = 34 | |  |  |  |  |  |  |  |
| VLMT 1-5 | 59.7 | (9.1) | 61.4 | (8.3) | 59.1 | (8.3) | 60.6 | (7.6) |  | -0.4 | [-3.3; 2.6] | 0.07 | 0.80 | <0.01 |  |
| VLMT 5-7 | 0.9 | (1.4) | 0.9 | (1.6) | 1.0 | (1.7) | 1.1 | (1.6) |  | 0.1 | [-0.7; 0.9] | 0.03 | 0.86 | <0.01 |  |
| SDMT (points) | 60.7 | (12.6) | 62.1 | (13.3) | 58.2 | (9.1) | 60.5 | (10.9) |  | 0.8 | [-2.4; 4.0] | 0.26 | 0.61 | <0.01 |  |
| BVMT-R total learning (points) | 25.4 | (5.7) | 24.4 | (5.7) | 26.4 | (6.2) | 26.0 | (5.5) |  | 1.1 | [-1.2; 3.4] | 0.86 | 0.36 | 0.02 |  |
| BVMT-R recall (points) | 9.7 | (2.2) | 9.9 | (1.7) | 10.0 | (2.0) | 9.8 | (2.0) |  | -0.4 | [-1.1; 0.4] | 0.92 | 0.34 | 0.02 |  |
| BVMT-R recognition hits (points) | 5.8 | (0.5) | 5.9 | (0.3) | 5.9 | (0.3) | 5.9 | (0.5) |  | 0.0 | [-0.3; 0.3] | 0.01 | 0.93 | <0.01 |  |
| BVMT-R false alarms (points) | 0.0 | (0.0) | 0.0 | (0.2) | 0.1 | (0.3) | 0.0 | (0.0) |  | 0.0 | [-0.1; 0.0] | 1.41 | 0.24 | 0.03 |  |
| TAP Tonic Alertness (msec) | 245.4 | (45.4) | 257.3 | (50.5) | 259.2 | (42.9) | 259.0 | (34.4) |  | -9.1 | [-22.0; 3.9] | 1.96 | 0.17 | 0.04 |  |
| TAP Phasic Alertness (msec) | 251.3 | (43.6) | 248.0 | (29.8) | 256.9 | (43.4) | 254.4 | (28.0) |  | 3.8 | [-7.1; 14.7] | 0.48 | 0.49 | 0.01 |  |
| TAP CSA valid (msec) | 290.1 | (48.3) | 288.7 | (47.0) | 301.6 | (44.4) | 295.9 | (41.5) |  | -0.1 | [-18.2; 18.0] | 0.00 | 0.99 | <0.01 |  |
| TAP CSA invalid (msec) | 329.6 | (58.8) | 325.4 | (50.5) | 348.4 | (58.6) | 345.8 | (62.6) |  | 7.9 | [-16.0; 31.9] | 0.44 | 0.51 | 0.01 |  |
| TAP Incompatibility (msec) | 487.4 | (65.8) | 492.0 | (60.4) | 488.0 | (76.9) | 485.8 | (69.7) |  | -6.5 | [-32.2; 19.2] | 0.26 | 0.61 | <0.01 |  |
| RWT verbal fluency I (points) | 24.4 | (7.7) | 18.1 | (6.0) | 24.3 | (7.7) | 18.8 | (6.9) |  | 0.7 | [-2.3; 3.7] | 0.22 | 0.64 | <0.01 |  |
| RWT verbal fluency II (points) | 36.7 | (9.5) | 27.8 | (7.6) | 36.6 | (9.6) | 27.6 | (7.3) |  | -0.1 | [-3.3; 3.0] | 0.01 | 0.94 | <0.01 |  |
| RWT verbal flexibilty (points) | 23.0 | (4.8) | 20.4 | (4.8) | 21.7 | (4.5) | 19.9 | (5.1) |  | 0.0 | [-2.5; 2.6] | 0.00 | 0.99 | <0.01 |  |
| PASAT (points) | 49.3 | (8.5) | 48.8 | (14.8) | 49.8 | (8.8) | 53.6 | (8.4) |  | 4.2 | [-0.1; 8.6] | 3.83 | 0.06 | 0.07 |  |
| Corsi forwards (points) | 9.4 | (2.4) | 9.9 | (2.2) | 8.8 | (1.9) | 9.6 | (1.9) |  | 0.1 | [-0.7; 0.9] | 0.04 | 0.84 | <0.01 |  |
| Corsi backwards (points) | 8.7 | (1.9) | 8.8 | (1.7) | 8.5 | (1.9) | 9.0 | (1.8) |  | 0.4 | [-0.3; 1.0] | 1.09 | 0.30 | 0.02 |  |
| MASC | 11.7 | (1.6) | 12.1 | (1.8) | 11.6 | (2.3) | 12.2 | (1.8) |  | 0.1 | [-0.7; 0.9] | 0.05 | 0.82 | <0.01 |  |
|  | | | | | | | | | | | | | | | |
