# Supplementary material for: Short-term interval aerobic exercise training does not improve memory functioning in relapsing-remitting multiple sclerosis—a randomized controlled trial
Source: PeerJ. 2018 Dec 12;6:e6037. doi: 10.7717/peerj.6037 (PMC6295157; doi:10.7717/peerj.6037)
Supplement: Supplemental Information 5 — Data as mean (standard deviation). IG: Intervention group; CG: Control group. 6 MWT: Six minute walking test; 9 HPT: Nine-Hole Peg Test; T25FW: Timed 25-foot walk; Pmax: maximal Power; IDS16-SR: 16-item version of Inventory of Depressive Symptomatology Self-Rated; FSMC: Fatigue Scale for Motor and Cognitive Functions; MSWS-12: 12-item MS Walking Scale; HAQUAMS: Hamburger Quality of Life Questionnaire in Multiple Sclerosis; *ANCOVA. [file peerj-06-6037-s005.docx]

|  | IG_PP | | | | | CG | | | | | |  | | Mean between group-difference  [95% CI] | | | f-value* | | p-value* | | Effect-size*  Partial eta sq | |  |  |
| --- | --- | --- | --- | --- | --- | --- | --- | --- | --- | --- | --- | --- | --- | --- | --- | --- | --- | --- | --- | --- | --- | --- | --- | --- |
|  | Baseline | | Week 12 | | | Baseline | | | Week 12 | | |  | |  |  |  |  |  |  |  |  |  |  |  |
|  | n = 23 | | n = 23 | | | n = 34 | | | n = 34 | | |  | |  |  |  |  |  |  |  |  |  |  |  |
| Motor function and aerobic fitness | | | | | | | | | | | | | | | | | | | | | | |  |  |
| 6 MWT (m) | 436.8 | (119.1) | | 461.8 | (107.6) | | 448.8 | (79.8) | | 466.7 | (84.2) | |  | | -0.1 | [-46.7; 46.4] | | 0.00 | | >0.99 | | <0.01 | |  |
| 9 HPT dominant (sec) | 18.8 | (2.5) | | 18.5 | (2.3) | | 19.1 | (2.9) | | 18.6 | (3.0) | |  | | -0.2 | [-0.9; 0.6] | | 0.21 | | 0.65 | | <0.01 | |  |
| 9 HPT non dominant (sec) | 19.2 | (2.2) | | 19.1 | (2.2) | | 19.8 | (4.1) | | 19.0 | (3.5) | |  | | -0.6 | [-1.5; 0.4] | | 1.43 | | 0.24 | | 0.03 | |  |
| T25FW (sec) | 4.6 | (0.8) | | 4.6 | (0.7) | | 4.8 | (0.8) | | 4.8 | (0.8) | |  | | 0.0 | [-0.2; 0.3] | | 0.09 | | 0.77 | | <0.01 | |  |
| V0_2peak_ (ml O_2_/min) | 2233.5 | (653.8) | | 2326.1 | (726.3) | | 1761.5 | (421.3) | | 1779.4 | (427.4) | |  | | -81.4 | [-218.4; 55.6] | | 1.42 | | 0.24 | | 0.03 | |  |
| V0_2peak_/kg ((ml O_2_/min)/kg) | 29.5 | (7.0) | | 30.4 | (7.6) | | 25.6 | (5.5) | | 25.6 | (5.4) | |  | | -1.5 | [-3.4; 0.5] | | 2.34 | | 0.13 | | 0.04 | |  |
| P_max_ (watt) | 168.0 | (43.7) | | 192.0 | (49.1) | | 139.5 | (31.1) | | 139.6 | (31.0) | |  | | -26.1 | [-38.0; -14.2] | | 19.23 | | <0.01 | | 0.27 | |  |
| Patient-reported outcome measures | | | | | | | | | | | | | | | | | | | | | | |  |  |
| IDS-16SR | 5.1 | (3.8) | | 5.0 | (3.5) | | 6.1 | (4.3) | | 6.3 | (4.6) | |  | | 0.6 | [-1.0; 2.2] | | 0.53 | | 0.47 | | 0.01 | |  |
| FSMC | 49.5 | (19.3) | | 48.2 | (19.7) | | 53.4 | (21.6) | | 50.9 | (21.4) | |  | | -0.7 | [-6.8; 5.4] | | 0.05 | | 0.82 | | <0.01 | |  |
| MSWS-12 | 16.4 | (7.3) | | 15.9 | (5.4) | | 18.7 | (10.7) | | 18.7 | (9.9) | |  | | 0.9 | [-1.1; 2.9] | | 0.83 | | 0.37 | | 0.02 | |  |
| HAQUAMS | 49.8 | (11.8) | | 49.7 | (12.9) | | 51.2 | (18.7) | | 51.8 | (14.7) | |  | | 1.1 | [-3.5; 5.6] | | 0.23 | | 0.63 | | <0.01 | |  |
|  | | | | | | | | | | | | | | | | | | | | | | | | |
