# Supplementary material for: Short-term interval aerobic exercise training does not improve memory functioning in relapsing-remitting multiple sclerosis—a randomized controlled trial
Source: PeerJ. 2018 Dec 12;6:e6037. doi: 10.7717/peerj.6037 (PMC6295157; doi:10.7717/peerj.6037)
Supplement: Supplemental Information 6 — mean values of raw scores (standard deviation). IG: Intervention group; CG: Control group. VLMT: Verbal Learning and Memory Test; SDMT: Symbol Digit Modalities Test; BVMT-R: Brief Visuospatial Memory Test-Revised; TAP Tonic Alertness: Test Battery for Attention Tonic Alertness; TAP Phasic Alertness: Test Battery for Attention Phasic Alertness; TAP CSA valid: Test Battery for Attention covert shift of attention valid; TAP CSA invalid: Test Battery for Attention covert shift of attention invalid; TAP Incompatibility: Test Battery for Attention Incompatibility; RWT: Regensburger Verbal Fluency Test; PASAT: Paced Auditory Serial Addition Test; MASC: Movie for Assessment of Social Cognition; *ANCOVA. [file peerj-06-6037-s006.docx]

|  | IG_RG | | | | CG | | | |  | Mean between group-difference  [95% CI] | | f-value* | p-value* | Effect-size*  Partial eta sq |  |
| --- | --- | --- | --- | --- | --- | --- | --- | --- | --- | --- | --- | --- | --- | --- | --- |
|  | Baseline | | Week 12 | | Baseline | | Week 12 | |  |  |  |  |  |  |  |
|  | n = 11 | | n = 11 | | n = 34 | | n = 34 | |  |  |  |  |  |  |  |
| VLMT 1-5 | 57.6 | (12.2) | 57.8 | (11.4) | 59.1 | (8.3) | 60.6 | (7.6) |  | 1.6 | [-2.2; 5.4] | 0.76 | 0.39 | 0.02 |  |
| VLMT 5-7 | 1.1 | (1.4) | 1.1 | (1.9) | 1.0 | (1.7) | 1.1 | (1.6) |  | 0.0 | [-1.0; 1.0] | 0.00 | 0.99 | <0.01 |  |
| SDMT (points) | 59.9 | (17.1) | 62.7 | (17.9) | 58.2 | (9.1) | 60.5 | (10.9) |  | -0.5 | [-5.1; 4.0] | 0.06 | 0.81 | <0.01 |  |
| BVMT-R total learning (points) | 26.4 | (7.1) | 25.1 | (7.0) | 26.4 | (6.2) | 26.0 | (5.5) |  | 0.9 | [-2.3; 4.1] | 0.32 | 0.58 | 0.01 |  |
| BVMT-R recall (points) | 10.3 | (2.3) | 10.6 | (1.6) | 10.0 | (2.0) | 9.8 | (2.0) |  | -0.6 | [-1.5; 0.4] | 1.60 | 0.21 | 0.04 |  |
| BVMT-R recognition hits (points) | 5.9 | (0.3) | 5.8 | (0.4) | 5.9 | (0.3) | 5.9 | (0.5) |  | 0.1 | [-0.3; 0.4] | 0.11 | 0.74 | <0.01 |  |
| BVMT-R false alarms (points) | 0.0 | (0.0) | 0.0 | (0.0) | 0.1 | (0.3) | 0.0 | (0.0) |  | 0.0 | [0.0; 0.0] | 0.00 | >0.99 | <0.01 |  |
| TAP Tonic Alertness (msec) | 258.1 | (54.9) | 259.1 | (58.2) | 259.2 | (42.9) | 259.0 | (34.4) |  | -0.9 | [-17.7; 16.0] | 0.01 | 0.92 | <0.01 |  |
| TAP Phasic Alertness (msec) | 263.6 | (54.8) | 243.8 | (37.8) | 256.9 | (43.4) | 254.4 | (28.0) |  | 13.9 | [-1.0; 28.7] | 3.56 | 0.07 | 0.08 |  |
| TAP CSA valid (msec) | 296.8 | (61.2) | 283.4 | (46.5) | 301.6 | (44.4) | 295.9 | (41.5) |  | 9.7 | [-12.5; 31.8] | 0.78 | 0.38 | 0.02 |  |
| TAP CSA invalid (msec) | 343.4 | (66.4) | 319.6 | (47.9) | 348.4 | (58.6) | 345.8 | (62.6) |  | 22.9 | [-8.3; 54.1] | 2.19 | 0.15 | 0.05 |  |
| TAP Incompatibility (msec) | 478.6 | (75.4) | 481.4 | (63.6) | 488.0 | (76.9) | 485.8 | (69.7) |  | -1.2 | [-37.1; 34.8] | 0.00 | 0.95 | <0.01 |  |
| RWT verbal fluency I (points) | 22.6 | (10.0) | 17.4 | (5.2) | 24.3 | (7.7) | 18.8 | (6.9) |  | 0.7 | [-3.2; 4.6] | 0.13 | 0.72 | <0.01 |  |
| RWT verbal fluency II (points) | 35.6 | (13.2) | 25.0 | (9.0) | 36.6 | (9.6) | 27.6 | (7.3) |  | 2.1 | [-1.7; 5.8] | 1.26 | 0.27 | 0.03 |  |
| RWT verbal flexibilty (points) | 22.4 | (5.5) | 19.8 | (5.5) | 21.7 | (4.5) | 19.9 | (5.1) |  | 0.3 | [-3.0; 3.7] | 0.04 | 0.84 | <0.01 |  |
| PASAT (points) | 45.0 | (9.5) | 44.7 | (18.9) | 49.8 | (8.8) | 53.6 | (8.4) |  | 4.7 | [-1.7; 11.2] | 2.20 | 0.15 | 0.05 |  |
| Corsi forwards (points) | 8.9 | (2.7) | 9.9 | (2.3) | 8.8 | (1.9) | 9.6 | (1.9) |  | -0.3 | [-1.3; 0.7] | 0.28 | 0.60 | 0.01 |  |
| Corsi backwards (points) | 8.3 | (2.3) | 8.6 | (2.2) | 8.5 | (1.9) | 9.0 | (1.8) |  | 0.3 | [-0.6; 1.3] | 0.52 | 0.48 | 0.01 |  |
| MASC | 11.6 | (1.5) | 12.6 | (1.2) | 11.6 | (2.3) | 12.2 | (1.8) |  | -0.4 | [-1.3; 0.5] | 0.75 | 0.39 | 0.02 |  |
|  | | | | | | | | | | | | | | | |
