# Supplementary material for: Short-term interval aerobic exercise training does not improve memory functioning in relapsing-remitting multiple sclerosis—a randomized controlled trial
Source: PeerJ. 2018 Dec 12;6:e6037. doi: 10.7717/peerj.6037 (PMC6295157; doi:10.7717/peerj.6037)
Supplement: Supplemental Information 7 — Data as mean (standard deviation). IG: Intervention group; CG: Control group. 6 MWT: Six minute walking test; 9 HPT: Nine-Hole Peg Test; T25FW: Timed 25-foot walk; Pmax: maximal Power; IDS16-SR: 16-item version of Inventory of Depressive Symptomatology Self-Rated; FSMC: Fatigue Scale for Motor and Cognitive Functions; MSWS-12: 12-item MS Walking Scale; HAQUAMS: Hamburger Quality of Life Questionnaire in Multiple Sclerosis; *ANCOVA. [file peerj-06-6037-s007.docx]

|  | IG_RG | | | | CG | | | |  | Mean between  group-difference  [95% CI] | | f-value* | | p-value* | | Effect-size*  Partial eta sq | |  |
| --- | --- | --- | --- | --- | --- | --- | --- | --- | --- | --- | --- | --- | --- | --- | --- | --- | --- | --- |
|  | Baseline | | Week 12 | | Baseline | | Week 12 | |  |  |  |  |  |  |  |  |  |  |
|  | n = 11 | | n = 11 | | n = 34 | | n = 34 | |  |  |  |  |  |  |  |  |  |  |
| Motor function and aerobic fitness | | | | | | | | | | | | | | | | | | |
| 6 MWT (m) | 430.6 | (121.2) | 461.7 | (95.1) | 448.8 | (79.8) | 466.7 | (84.2) |  | 1.7 | [-59.0; 62.4] | | 0.00 | | 0.95 | | <0.01 |  |
| 9 HPT dominant (sec) | 18.3 | (2.0) | 18.4 | (2.6) | 19.1 | (2.9) | 18.6 | (3.0) |  | -0.5 | [-1.5; 0.5] | | 1.02 | | 0.32 | | 0.02 |  |
| 9 HPT non dominant (sec) | 18.5 | (1.5) | 18.7 | (2.2) | 19.8 | (4.1) | 19.0 | (3.5) |  | -0.7 | [-2.1; 0.7] | | 0.89 | | 0.35 | | 0.02 |  |
| T25FW (sec) | 4.5 | (0.8) | 4.5 | (0.7) | 4.8 | (0.8) | 4.8 | (0.8) |  | 0.0 | [-0.4; 0.4] | | 0.04 | | 0.85 | | <0.01 |  |
| V0_2peak_ (ml O_2_/min) | 2415.5 | (647.2) | 2660.0 | (653.3) | 1761.5 | (421.3) | 1779.4 | (427.4) |  | -287.5 | [-468.1; -106.8] | | 10.33 | | <0.01 | | 0.20 |  |
| V0_2peak_/kg ((ml O_2_/min)/kg) | 28.5 | (6.4) | 31.6 | (7.1) | 25.6 | (5.5) | 25.6 | (5.4) |  | -3.6 | [-5.9; -1.2] | | 8.98 | | <0.01 | | 0.18 |  |
| P_max_ (watt) | 169.6 | (41.0) | 214.4 | (42.7) | 139.5 | (31.1) | 139.6 | (31.0) |  | -48.8 | [-62.6; -35.1] | | 51.28 | | <0.01 | | 0.56 |  |
| Patient-reported outcome measures | | | | | | | | | | | | | | | | | | |
| IDS-16SR | 4.5 | (4.8) | 3.8 | (2.1) | 6.1 | (4.3) | 6.3 | (4.6) |  | 1.4 | [-0.7; 3.5] | | 1.75 | | 0.19 | | 0.04 |  |
| FSMC | 42.3 | (16.5) | 39.9 | (14.3) | 53.4 | (21.6) | 50.9 | (21.4) |  | 1.5 | [-5.6; 8.7] | | 0.18 | | 0.67 | | <0.01 |  |
| MSWS-12 | 15.4 | (9.2) | 14.3 | (3.9) | 18.7 | (10.7) | 18.7 | (9.9) |  | 1.8 | [-0.8; 4.5] | | 1.92 | | 0.17 | | 0.04 |  |
| HAQUAMS | 46.3 | (7.6) | 45.3 | (7.2) | 51.2 | (18.7) | 51.8 | (14.7) |  | 3.4 | [-2.5; 9.2] | | 1.37 | | 0.25 | | 0.03 |  |
|  | | | | | | | | | | | | | | | | | | |
